# Assessing Genotoxicity of Bovine Pericardium in Guided Tissue Regeneration: Bacterial Reverse Mutation Assay With Exogenous Metabolic Activation

**DOI:** 10.7759/cureus.64078

**Published:** 2024-07-08

**Authors:** Abdo Mohammed Mohammed Abdulrazzaq

**Affiliations:** 1 Department of Preventive Dentistry, Faculty of Dentistry, Najran University, Najran, SAU

**Keywords:** regeneration, guided tissue, bovine pericardium, genotoxicity, assessing

## Abstract

Introduction

Guided tissue regeneration (GTR) is integral to periodontal therapy, facilitating the repair of osseous defects. Due to the widespread use of bovine pericardium (BP) in GTR, a thorough investigation into its genotoxicity is essential for patient safety and treatment efficacy. This study aimed to evaluate the genotoxic effects of local BP in GTR for periodontal osseous defects.

Materials and methods

The Bacterial Reverse Mutation Assay (Ames test) was used to assess the genotoxic potential of local BP. An exogenous metabolic activation system was employed to evaluate the direct effects of the material on bacterial cells.

Results

The study investigated the mutagenic effects of local BP across multiple strains of *Salmonella typhimurium*, utilizing concentrations ranging from 0.3125 mg/plate to 5 mg/plate. While some variability was observed in revertant counts, the generally low SDs suggest a consistent response to the test substance. The maximum revertant count for each strain did not significantly exceed the mean values, indicating the absence of notable outliers or exceptionally high revertant counts at any specific concentration. Based on the data and toxicity assessment criteria, there is insufficient evidence to suggest that the experimental material induces genotoxic effects in the tested bacterial strains under the provided experimental conditions.

Conclusion

This study assessed the mutagenic potential of local BP membranes used in GTR with the Ames test. Results showed no evidence of mutagenicity, as revertant counts did not exceed twice the negative control in all bacterial strains with exogenous metabolic activation. This suggests that bovine pericardium membranes are safe for medical use under the test conditions. The study highlights the biocompatibility and non-mutagenic nature of BP membranes in GTR for periodontal therapy.

## Introduction

The evolution of biocompatible materials has emerged from extensive multidisciplinary research efforts. Material selection is guided by various criteria, including toxicology, biocompatibility, and biodegradability. The primary objectives in biomaterial development are to guarantee the quality and safety of the final product, aligning with regulatory standards [1]. Resorbable membranes were developed to address issues with non-degradable membranes made from polyglycoside synthetic polymers, collagen, and calcium sulfate; they offer better tissue compatibility. These membranes do not require a second surgery for removal and allow regulated bio-absorption, simplifying clinical management [2].

The researchers explored the potential uses of pericardium membranes in guided bone regeneration (GBR), with a focus on highlighting their advantages in dentistry, especially in implantology. The study underscored the extended barrier functionality and potential benefits of these membranes compared to native collagen membranes [3]. The researchers concentrated on regenerative periodontal therapy. Their aim was to investigate the viability of the pericardium membrane in restoring tooth-supporting tissues lost because of inflammatory periodontal disease. Noted for its biocompatibility and efficacy in tissue repair, the pericardium membrane demonstrated potential as an appropriate material for guided tissue regeneration (GTR) [4]. The researchers also examined a collagen membrane derived from porcine pericardium for GBR in minipigs. Their aim was to evaluate its resorption kinetics and barrier efficacy. The findings indicated that the pericardium-derived membrane was statistically comparable to the control membrane, suggesting the need for additional clinical assessment in GBR procedures [5].

Biocompatibility assessment is a pivotal element in the development of GTR materials, focusing on both biosafety and bio-functionality. Biosafety encompasses evaluations of cytotoxicity as well as more intricate considerations such as mutagenesis and carcinogenesis. Bio-functionality pertains to a material's performance and its interaction with host tissues. A variety of in vitro and in vivo tests are utilized to evaluate these aspects, adhering to standards such as ISO 10993-3 for assessing genotoxicity, carcinogenicity, and reproductive toxicity [6]. Efforts to develop and validate protocols aimed at enhancing the biocompatibility and structural integrity of bovine bone and pericardium pave the way for improved applications in bone grafting and GBR procedures. These findings not only enrich the current understanding but also offer promising prospects for future clinical utilization in oral surgery and dental implantology [7].

The researchers sought to develop and characterize decellularized and glutaraldehyde-crosslinked bovine pericardium (GC-BP) for GBR. Their aim was to evaluate the applicability of GC-BP as a scaffold. The investigation revealed that GC-BP displayed conformational alterations in collagen molecules, leading to reduced mechanical properties while enhancing cell adhesion and viability of dental pulp stem cells. These findings suggest that GC-BP holds promise as a potential candidate for GBE [8]. Genetic toxicity testing is crucial for assessing the safety of materials employed in GTR. This encompasses a range of assays aimed at detecting DNA damage, gene mutations, and chromosomal abnormalities. The International Organization for Standardization (ISO) underscores the necessity of these evaluations for medical devices, particularly those intended for prolonged contact with the body [9]. The researchers conducted a study to evaluate the cytotoxicity and genotoxicity of BP preserved in glycerol, aiming to assess the potential toxicity of this commonly used biomaterial. The pericardium was prepared without prior washing and sterilized using gamma radiation. It was then immersed in RPMI 1640 culture medium, and the same extract was utilized for both cytotoxic and genotoxic tests on Chinese hamster ovary cells. The findings revealed that while the pericardium preserved in glycerol exhibited some cytotoxic effects, it did not demonstrate any genotoxicity [10].

## Materials and methods

Study design

This experimental study was designed to assess mutations using a bacterial reverse mutation assay with metabolic activation systems, under the influence of the test substance (BP). The primary assessment focused on counting the number of revertant colonies, serving as an indicator of the biocompatibility of the test substance. The study was conducted at the Ames Test Laboratory, School of Dental Sciences, Universiti Sains Malaysia (USM).

Experimental material (local BP)

The experimental material used was local BP from the National Tissue Bank at USM. BP is primarily composed of collagen fibers and is recognized for its versatility as a natural biological membrane. It is easy to handle, flexible, adaptable, remodels well, and is resilient, making it suturable. It also comes in different configurations. BP was stored aseptically at room temperature. Due to the storage conditions, sterilization, and local manufacturing processes, assessing its genotoxicity potential was necessary to ensure BP is safe for medical uses, especially in surgical procedures requiring GTR.

Positive controls

Positive controls used in the study included 4-Nitro-O-phenylenediamine, Sodium azide, Acridine orange, and 2-Aminoanthracene, obtained from different manufacturers. These controls were stored under specified conditions and handled with aseptic precautions to maintain test accuracy.

Bacterial cultures

Selection and Preservation of Bacterial Cultures

**Figure 1:**
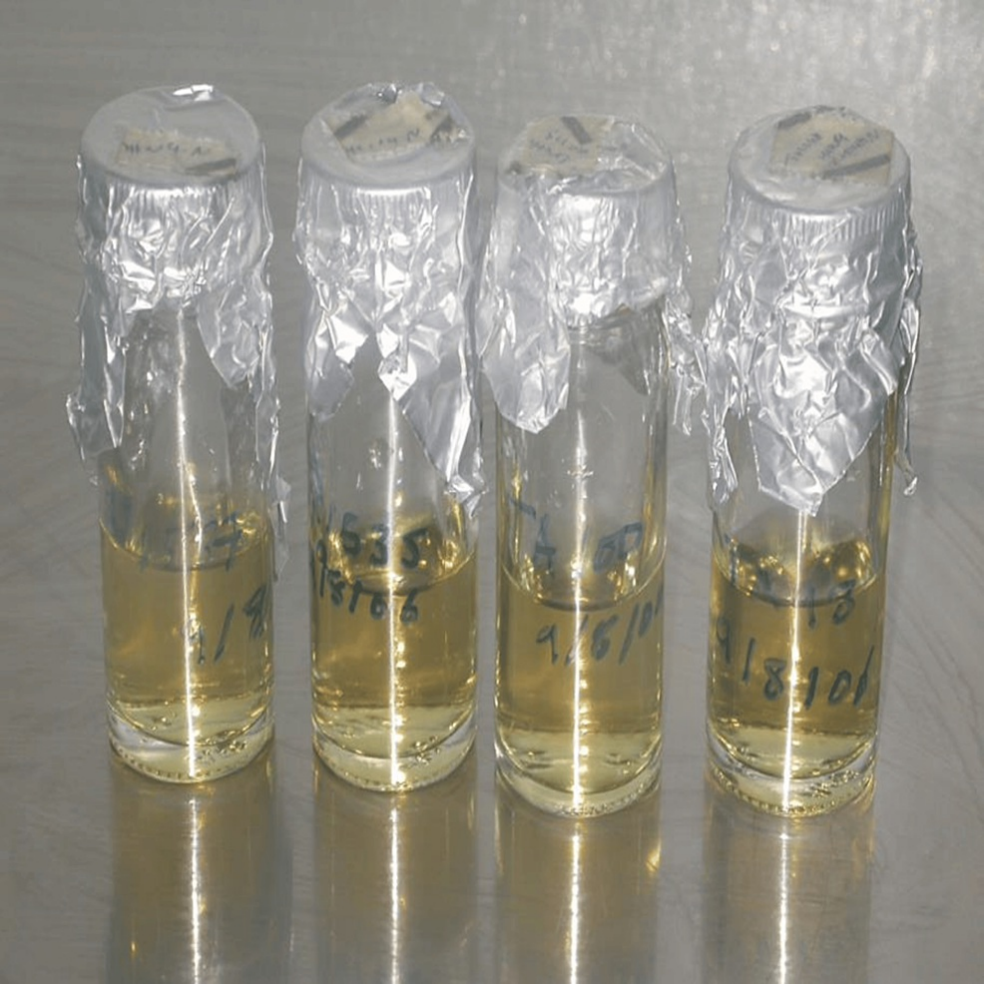
Bacterial cultures.

Salmonella typhimurium strains TA 1535, TA 1537, TA 98, and TA100 were employed for detecting base-pair substitution and frameshift mutations. These strains were preserved at -80°C in an ultra-deep freezer, as shown in Figure 1.

Characterization of the Strains

The characteristics of each strain, including mutations affecting amino acid synthesis, DNA repair, membrane mutation, and the presence of R-factor, are shown in Table 1.

**Table 1:** Characteristics of the strains.

Strains (Salmonella typhimurium)	Mutation on synthesis of amino acid	Mutation on excision repair	Membrane mutation (LPS)	R-Factor (PKM101)
TA 98	hisD3052	∆ uvrB	rfa	+
TA 100	hisG46	∆ uvrB	rfa	+
TA 1535	hisG46	∆ uvrB	rfa	_
TA 1537	hisC3076	∆ uvrB	rfa	_

Reagents and medium

Reagents

A variety of reagents were utilized in the study, including Vogel-Bonner Salts, glucose solution, histidine/biotin solution, top agar, nutrient broth, an exogenous metabolic activation system, enriched glucose minimal (GM) agar plates, biotin and histidine solutions, ampicillin solution, crystal violet solution, and nutrient agar plates.

Medium and S9 Mix

In the study, GM agar and soft agar were utilized, along with an S9 homogenate (liver microsomal enzymes), in the Ames test.

Experimental Material Preparation

BP) was extracted in sterile water and subsequently incubated prior to undergoing mutagenicity testing using the standard plate incorporation assay.

Positive Controls Preparation

The positive controls were dissolved in distilled water and then stored at -80°C.

Methodology

The Ames test was conducted using the pre-incubation method for bacterial strains TA 98, TA 100, TA 1535, and TA 1537, with an exogenous metabolic activation system. Testing involved triplicates for negative controls and duplicates for test substances and positive controls.

Procedures

The Ames test, initially developed by Ames, was employed to detect a broad spectrum of genetic damage leading to mutations. In the pre-incubation assay, a variation of the standard plate incorporation method, tester strains were exposed to the test agent for 20 minutes in a small volume before being plated on GM agar medium. After an incubation period at 37 ± 0.5 ºC for 48 hours, revertant colonies were enumerated [11].

Pure water was utilized as a negative control, while various chemical agents served as positive controls for each bacterial strain, as shown in Table 2.

**Table 2:** Positive controls of bacterial strains.

	TA 100	TA 1535	TA 98	TA 1537
S9 Mix (+)	NaN3	NaN3	4NOP	AO
	5 ug/plate	2.5 ug/plate	2.5 ug/plate	50 ug/plate

Selection of Dosages

Doses ranging from 0.3125 to 5 mg per plate were examined, and no discernible growth inhibition was observed across all bacterial tester strains when utilizing an exogenous metabolic activation system. Additionally, there was no significant increase in revertant colonies at any of the tested doses for all tester strains.

Microscopic Observation

The state of the revertant colonies, including their size and number, as well as any growth inhibition, were examined using a light microscope.

Counting of Bacterial Colonies

**Figure 2:**
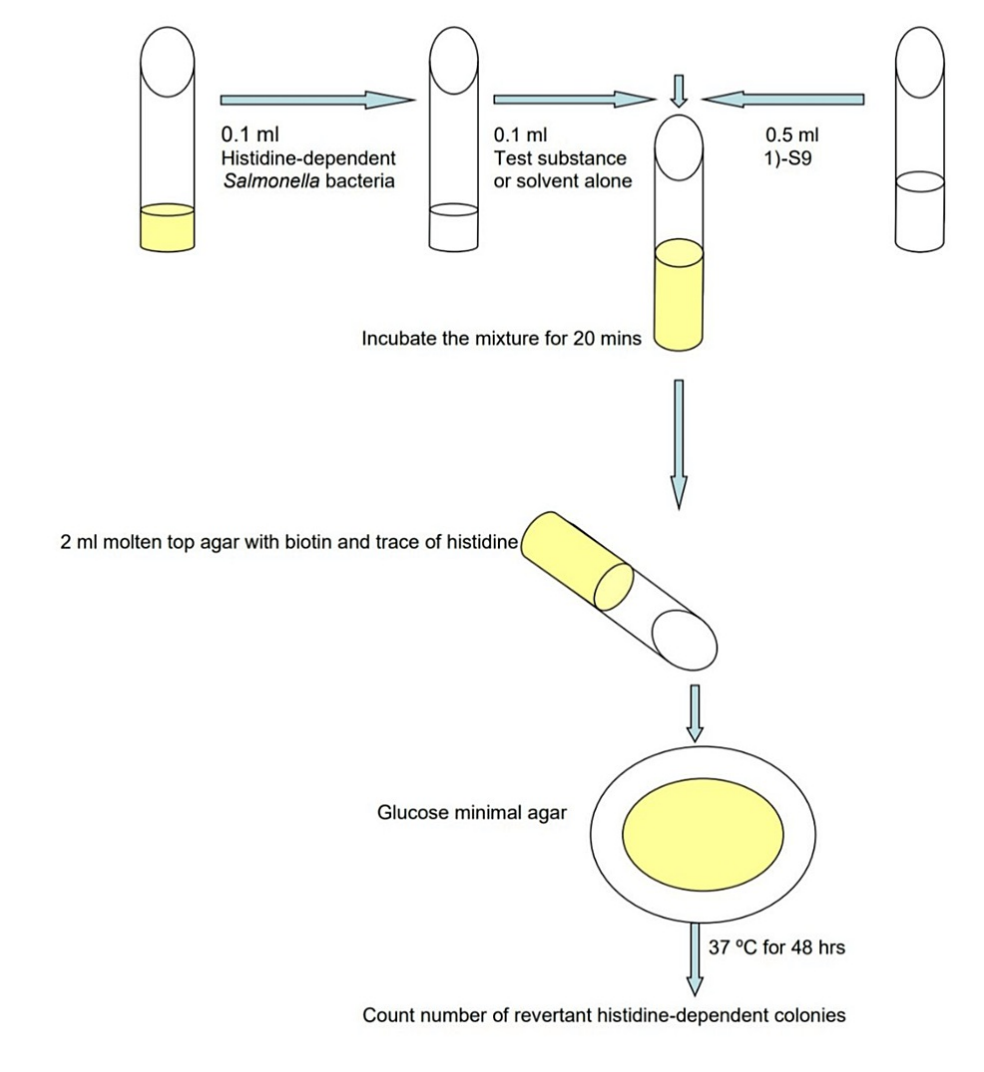
Ames test procedure.

Bacterial colonies were counted either manually or using a colony counting device. Each plate was enumerated three times, and the mean value was used for the final count. The average number of revertants per dose was calculated based on these counts, as shown in Figure 2.

## Results

The test material was classified as negative if the number of revertant colonies was less than double that of the negative control. This section analyzes toxicity indicators and growth inhibition in all bacterial tester strains. The procedure provides a thorough framework for evaluating the genotoxicity of local BP. Standardized protocols, specific bacterial strains, and precise dosing regimens are used. These measures enhance the trustworthiness and precision of the test outcomes, which is crucial for assessing the safety and compatibility of biomaterials, as shown in Table 3.

**Table 3:** Criterion toxicity assessment.

Strain	Double Negative Control Count	Is Mean Count > Double Negative?
TA 98	678	No
TA 1537	912	No
TA 100	586	No
TA 1535	890	No

Descriptive statistics for revertant counts across different concentrations of a test substance are provided for four bacterial strains: TA 98, TA 1537, TA 100, and TA 1535. Analyzing these statistics offers insights into the response of each strain to the test substance, as detailed in Table 4.

**Table 4:** Descriptive statistics for revertant counts across different concentrations of test substance.

Statistical Measure	TA 98	TA 1537	TA 100	TA 1535
Mean	255.2	283.4	258.2	356.6
Median	248	286	277	333
SD	40.48	44.5	56.02	74.76
Minimum	211	227	159	305
Maximum	300	337	294	486
Range	89	110	135	181

For TA 98, the average revertant count is noted at 255.2, with a median of 248, suggesting a central tendency around these values. The standard deviation, a measure of variability, is 40.48, indicating some spread in the data. The minimum and maximum revertant counts for this strain are 211 and 300, respectively, with a range of 89, showing the extent of variability in the responses.

TA 1537 exhibits an average revertant count of 283.4, slightly higher than TA 98, with a median very close to the mean at 286, suggesting a relatively symmetric distribution of data. The variability for TA 1537, as indicated by a standard deviation of 44.5, and a range of 110 between the minimum and maximum revertant counts (227 and 337), points to moderate variability in how this strain responds to the test substance.

TA 100’s average revertant count is 258.2, with a median of 277, which is notably higher than the mean, indicating the presence of lower outliers affecting the distribution. This strain shows the highest variability among the four, with a standard deviation of 56.02 and a range of 135, stretching from a minimum of 159 to a maximum of 294 revertant counts. This significant variation suggests a wide range of responses to the test substance.

Lastly, TA 1535 shows the highest response to the test substance, with an average revertant count of 356.6, substantially higher than the other strains. The median revertant count for TA 1535 is 333, indicating a distribution skewed towards higher counts. The variability within this strain’s response is also considerable, with a standard deviation of 74.76 and a range of 181, spanning from 305 to 486 revertant counts. Overall, the analysis reveals that TA 1535 is the most responsive strain to the test substance, exhibiting both the highest average revertant count and the greatest variability. In contrast, TA 98 appears to be the least responsive.

The SD and range values across the strains highlight the diversity in response to the test substance, with TA 100 and TA 1535 showing substantial variability. The closeness of the mean and median values for each strain generally suggests symmetric distributions of revertant counts, except for TA 100, where the distribution may be slightly skewed due to the influence of lower outliers. This comprehensive overview underscores the nuanced response of each strain to the test substance, indicating a complex interaction between the bacterial strains and the concentrations of the test substance.

This analysis indicates that for none of the strains (TA 98, TA 1537, TA 100, TA 1535), does the mean number of revertants per plate at any concentration of the test material exceed double the count of the negative control. Therefore, based on this criterion, the substance does not indicate toxicity in the context of the question, which depends on whether the count is greater than double the negative control to determine toxicity.

The revised visualizations present bar charts for each bacterial strain (TA 98, TA 1537, TA 100, TA 1535), showing the mean revertant counts at each test concentration. The red dashed line across each chart represents the threshold of double the negative control's revertant count, serving as a criterion for assessing potential toxicity.

Here’s an explanation of the key components and insights that can be drawn from the diagram below:

Key observations

Variability Across Concentrations

The mean revertant counts vary across concentrations for each strain, which is expected in dose-response studies.

Comparison to Toxicity Threshold

The red dashed line (double the negative control count) serves as a benchmark for toxicity assessment. None of the strains at any concentration show mean revertant counts exceeding this threshold significantly, aligning with our earlier analysis that there is no clear indication of toxicity based on this criterion.

Strain-Specific Responses

The charts also highlight the strain-specific responses to the test substance, underscoring the importance of using multiple strains in mutagenicity testing to capture a range of possible reactions.

**Figure 3:**
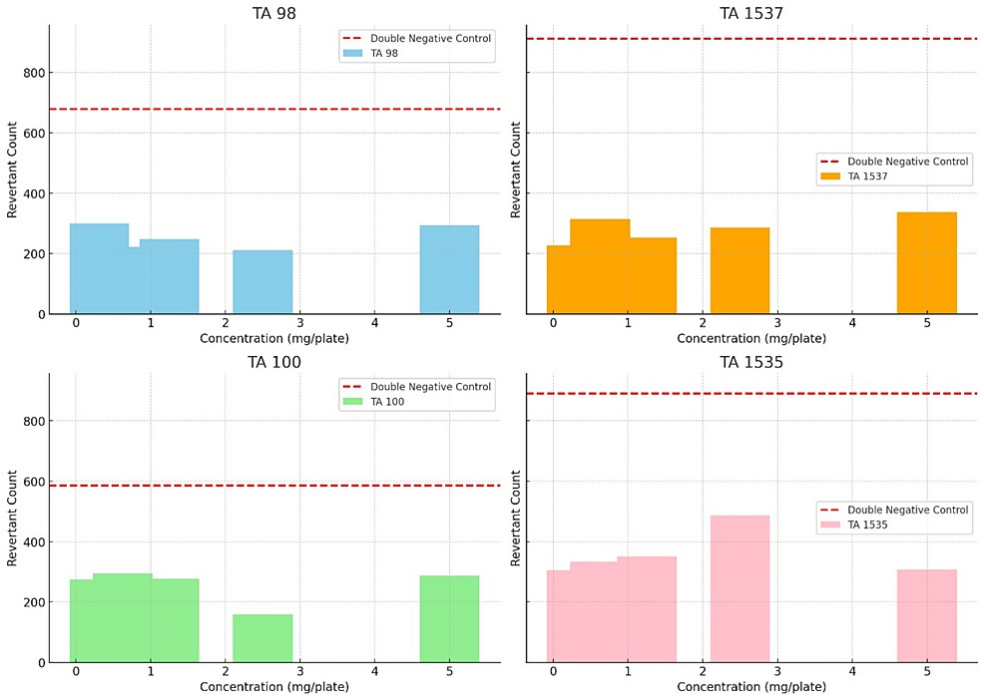
Charts highlighting strain-specific responses to the test substance.

These visualizations provide a clear and direct comparison of the test results against the specified toxicity assessment criterion, supporting the conclusion that, under the conditions tested, the substance does not demonstrate toxicity according to the criterion of causing revertant counts more than double those of the negative control, as shown in Figure 3.

The table below clearly indicates that for none of the strains (TA 98, TA 1537, TA 100, TA 1535) at any concentration (0.3125, 0.625, 1.25, 2.5, 5 mg/plate) did the mean revertant count in the test exceed double the mean revertant count of the negative control. Thus, according to the criterion used for this analysis, the test substance would not be considered toxic in terms of inducing a significant increase in the number of bacterial colonies compared to the negative control, as shown in Table 5.

**Table 5:** The number of bacterial colonies of the test substance compared to the negative control.

Strain	Concentration (mg/plate)	Mean Rev. Count (Test)	Double Mean Rev. Count (Control)	Toxicity Assessment
TA 98	0.3125	300	678	No
TA 98	0.625	223	678	No
TA 98	1.25	248	678	No
TA 98	2.5	211	678	No
TA 98	5	294	678	No
TA 1537	0.3125	227	912	No
TA 1537	0.625	314	912	No
TA 1537	1.25	253	912	No
TA 1537	2.5	286	912	No
TA 1537	5	337	912	No
TA 100	0.3125	274	586	No
TA 100	0.625	294	586	No
TA 100	1.25	277	586	No
TA 100	2.5	159	586	No
TA 100	5	287	586	No
TA 1535	0.3125	305	890	No
TA 1535	0.625	333	890	No
TA 1535	1.25	351	890	No
TA 1535	2.5	486	890	No
TA 1535	5	308	890	No

## Discussion

Extensive testing is imperative to assess the cytotoxic, mutagenic, or carcinogenic properties of new materials before their approval for medical use. These rigorous evaluations are crucial to guarantee the safety and efficacy of biomaterials in various medical applications. To enhance the efficiency of biocompatibility assessment, it is now advised that new biomaterials undergo initial in vitro cytotoxicity and mutagenicity testing. This recommendation holds particular significance for both temporary and permanent implants and prostheses, as the potential for carcinogenesis often arises from prolonged exposure to low concentrations of mutagenic agents [12]. The bacterial reverse mutation test, commonly referred to as the Ames test, evaluates the mutagenic properties of substances in vitro, employing exogenous metabolic activation. However, it is crucial to note that these in vitro metabolic activation systems cannot entirely mimic the complexities of in vivo mammalian conditions. As a result, the findings of the test may not directly correlate with the mutagenic or carcinogenic potential observed in mammals [13].

We conducted the Salmonella/microsome assay, commonly known as the Ames test, to evaluate the genotoxicity of BP. Designed for rapid screening with minimal test material, this test provides valuable insights. However, it is essential to recognize that it employs prokaryotic cells, which differ significantly from mammalian cells. Our focus in this study was on assessing the genotoxic potential of local BP membrane, which is utilized in GTR. The objective was to ascertain the mutagenic properties of BP. Our findings indicate the non-genotoxic nature of the BP membrane, as determined by the metabolic Salmonella/microsome assay (Ames test). This assay enables swift screening for genotoxic effects using a minimal amount of test material [14]. In our study, a notable concentration-dependent increase of at least 2-fold in the mean colonies per plate was observed for at least one tester strain, surpassing the mean colonies per plate for the relevant vehicle count [15]. Pure water was utilized as the negative control in our investigation. The outcomes obtained with metabolic activation for all four tester strains were consistent with the background data from our laboratory, thus affirming the reliability of the bacterial strains employed.

Researchers focused on regenerative periodontal therapy, exploring the potential of the pericardium membrane in reestablishing tooth-supporting tissues lost to inflammatory periodontal disease. Their findings suggest that the pericardium membrane, with its biocompatibility and effectiveness in tissue repair, is a promising material for GTR [4]. In our assessment of the local BP membrane as a prospective biomaterial for GTR, we emphasized the critical aspect of biocompatibility, a fundamental requirement for any biomaterial intended for clinical use. Our investigation involved the utilization of BP processed through a standardized procedure, comprising thorough cleaning, solvent dehydration, and sterilization via gamma irradiation. The principal objective was to evaluate the genotoxic potential of this local BP product, serving as the test substance in our in vitro experimentation. The biomaterial employed was in the form of a solid membrane, underscoring its practical suitability for medical applications. In our investigation, we followed established guidelines for the Ames test. Based on cytotoxicity assessments [13], we determined the maximum test concentration of BP to be 5 mg/plate or 5 µl/plate. The test was performed using an exogenous metabolic activation system.

The incubation temperature was maintained at 37°C, approximating the temperature of the human body, which is pertinent for assessing the bioabsorbable properties of BP utilized in GTR [16]. We selected an incubation period of 24 hours, consistent with the standard extraction time advocated by the majority of authors [17]. Mutagenic substances have the capability to induce reversion in histidine-deficient strains, enabling their growth and colony formation in a histidine-limited medium, unlike non-reverted strains. In this study, we used four distinct strains to evaluate various genomic mutations, including frameshift mutations (TA 98 and TA 1537) and base substitutions (TA 100 and TA 1535) [18]. However, the limitations of the study include that an in vitro genotoxicity test using mammalian cells was not conducted and must be carried out in the future. Additionally, an in vivo genotoxicity test has not been performed but should be undertaken if scientifically indicated or if in vitro results suggest potential genotoxicity.

## Conclusions

This study aimed to assess the mutagenic potential of BP membranes, commonly utilized in GTR. Using the Ames test, our investigation concluded that there was no evidence of mutagenicity under the experimental conditions. This was evidenced by revertant counts not exceeding twice the negative control count in all bacterial tester strains with the exogenous metabolic activation system, suggesting the non-mutagenicity of BP membranes. The implications of these findings are significant in the realm of tissue regeneration and biomaterial safety. They contribute valuable insights, supporting the safe utilization of BP membranes in medical applications. However, it is essential to acknowledge that these results are specific to the conditions under which the tests were conducted. Therefore, caution should be exercised in extrapolating these findings without further research. In summary, our study contributes to the expanding body of evidence affirming the biocompatibility of BP membranes, bolstering their viability as a secure option in GTR. The absence of mutagenic effects under the tested conditions provides reassurance regarding their suitability for medical use. However, continuous evaluation is advised to ensure their safety across diverse applications.

## References

[1] Grosskinsky U (2006). Biomaterial regulations for tissue engineering. Desalination J.

[2] Kao RT, Conte G, Nishimine D, Dault S (2005). Tissue engineering for periodontal regeneration. J Calif Dent Assoc.

[3] Gupta S, Gupta R (2014). Guided bone regeneration with Pericardium membranes. IOSR J Dent Med Sci.

[4] Setyawati EM, Klana NAP (2020). Concise review: periodontal tissue regeneration using pericardium membrane as guided bone regeneration. AIP Conf Proceed.

[5] Bornert F, Herber V, Sandgren R, Witek L, Coelho PG, Pippenger BE, Shahdad S (2021). Comparative barrier membrane degradation over time: pericardium versus dermal membranes. Clin Exp Dent Res.

[6] Kirkpatrick CJ, Bittinger F, Wagner M, Köhler H, van Kooten TG, Klein CL, Otto M (1998). Current trends in biocompatibility testing. Proc Inst Mech Eng H.

[7] Gardin C, Ricci S, Ferroni L (2015). Decellularization and delipidation protocols of bovine bone and pericardium for bone grafting and guided bone regeneration procedures. PLoS One.

[8] Ordóñez-Chávez GD, Rodríguez-Fuentes N, Peñaloza-Cuevas R (2023). In vitro evaluation of crosslinked bovine pericardium as potential scaffold for the oral cavity. Biomed Mater Eng.

[9] International Organization for Standardization (ISO) 10993-3 (2014). Biological evaluation of medical devices - Part 3: Tests for genotoxicity, carcinogenicity and reproductive toxicity. ISO.

[10] Rodas AC, Maizato MJ, Leirner AA, Pitombo RN, Polakiewicz B, Beppu MM, Higa OZ (2008). Cytotoxicity and genotoxicity of bovine pericardium preserved in glycerol. Artif Organs.

[11] Ames BN, McCann J, Yamasaki E (1975). Method for detecting carcinogens and mutagens with the Salmonella/Mammalian - microsome mutagenicity test. Mutation Res.

[12] Katzer A, Marquardt H, Westendorf J, Wening JV, Foerster G (2002). Polyetheretherketone--cytotoxicity and mutagenicity in vitro. Biomaterials.

[13] Organization for Economic Co-operation and Development (OECD) (2020). Bacterial reverse mutation test. OECD.

[14] Sawi J, Kanou F, Igrashi H (1995). Evaluation of growth inhibitory effect of ceramics powder slurry on bacteria by conductance method. J Chem Eng Jpn.

[15] Cariello NF, Piegorsch WW (1996). The Ames test: the two-fold rule revisited. Mutat Res/Gen Toxicol.

[16] Wening JV, Marquardt H, Katzer A (1995). Cytotoxicity and mutagenicity of Kevlar: an in vitro evaluation. Biomaterials.

[17] Maron DM, Ames BN (1983). Revised methods for the Salmonella mutagenicity test. Mutat Res.

[18] Mortelmans K, Zeiger E (2000). The Ames Salmonella/ microsome mutagenicity assay. Mutat Res.

